# Anti-Photoaging Effects of Low Molecular-Weight Fucoidan on Ultraviolet B-Irradiated Mice

**DOI:** 10.3390/md16080286

**Published:** 2018-08-18

**Authors:** Young-In Kim, Won-Seok Oh, Phil Hyun Song, Sungho Yun, Young-Sam Kwon, Young Joon Lee, Sae-Kwang Ku, Chang-Hyun Song, Tae-Ho Oh

**Affiliations:** 1 Department of Veterinary Internal Medicine, College of Veterinary Medicine, Kyungpook National University, Daegu 41566, Korea; kimyoungin@kpclab.co.kr (Y.-I.K.); owsvcs@hanmail.net (W.-S.O.); 2 KPC Corporation, Gwangju 12773, Korea; 3 Department of Urology, College of Medicine, Yeungnam University, Daegu 42415, Korea; sph04@yu.ac.kr; 4 Department of Veterinary Surgery, College of Veterinary Medicine, Kyungpook National University, Daegu 41566, Korea; shyun@knu.ac.kr (S.Y.); kwon@knu.ac.kr (Y.-S.K.); 5 Department of Preventive Medicine, College of Korean Medicine, Daegu Haany University, Gyeongsan 38610, Korea; gksxntk@dhu.ac.kr; 6 Department of Anatomy and Histology, College of Korean Medicine, Daegu Haany University, Gyeongsan 38610, Korea; gucci200@hanmail.net

**Keywords:** skin-aging, UVB, low molecular-weight, fucoidan, antioxidant, anti-inflammation, MMP

## Abstract

Ultraviolet (UV) B exposure induces DNA damage and production of reactive oxygen species (ROS), which causes skin photoaging through signaling pathways of inflammation and modulation of extracellular matrix remodeling proteins, collagens, and matrix metalloproteinase (MMP). As low molecular-weight fucoidan (LMF) has potential antioxidant and anti-inflammatory properties, we examined the protective effects of LMF against UVB-induced photoaging. A UVB-irradiated mouse model was topically treated with myricetin or LMF at 2.0, 1.0 and 0.2 mg/cm^2^ (LMF2.0, LMF1.0 and LMF0.2, respectively) once a day for 15 weeks. Wrinkle formation, inflammation, oxidative stress, MMP expression, and apoptosis in the treated regions were compared with those in a distilled water-treated photoaging model (UVB control). LMF treatments, particularly LMF2.0 and LMF1.0, significantly inhibited the wrinkle formation, skin edema, and neutrophil recruitment into the photo-damaged lesions, compared with those in the UVB control. While LMF decreased interleukin (IL)-1β release, it increased IL-10. The LMF treatment inhibited the oxidative stresses (malondialdehyde and superoxide anion) and enhanced endogenous antioxidants (glutathione). Additionally, LMF reduced the mRNA expression of MMP-1, 9, and 13. The histopathological analyses revealed the anti-photoaging effects of LMF exerted via its antioxidant, anti-apoptotic, and MMP-9-inhibiting effects. These suggest that LMF can be used as a skin-protective remedy for photoaging.

## 1. Introduction

Along with the increasing aging population and their demands for maintaining youthful skin, a development of skin anti-aging agents has attracted attention in the pharmaceutical and cosmetic science fields. Skin aging is divided into intrinsic chronological aging and extrinsic aging caused by various external stimuli, mainly ultraviolet (UV) radiation, called photoaging. In particular, UVB comprising 5–10% of all UV wavelengths is considered as the main cause of skin photoaging characterized by wrinkles, thickness, laxity, roughness, and pigmentation [1]. The mechanism involves a direct DNA damage and formation of photoproducts including cyclobutane pyrimidine dimers and pyrimidine (1,2) pyrimidine photoproducts. The photoproducts trigger apoptosis, cytokine release, immunosuppression, and signal transduction, severely followed by carcinogenesis [2]. In addition, repetitive exposure to UVB increases intracellular reactive oxygen species (ROS), leading to oxidative DNA damage, and activation of inflammation and extracellular matrix (ECM) remodeling proteins including matrix metalloproteinases (MMPs) [3]. Currently, UVB-induced photoaging has recently increased because of progressive depletion of the ozone layer, and thus optimal anti-photoaging remedies are required to increase treatment options.

Basic photoaging prevention is simply blocking sunlight through protective clothing or filters. However, because UVB exposure has positive effects on vitamin (Vt.) D3 synthesis, particularly in chronic kidney disease patients, the strict photoprotection may need additional vitamin (Vt.) D supplementation [4]. Additionally, the method is not effective for treating skin that is already photo-damaged. Thus, many topical treatments have been evaluated to reduce photoaging; retinoids, known as Vt. A (i.e., tretinoin, tazarotene, adapalene, retinol and retinaldehyde, alitretinoin), are drugs shown to reverse skin aging. However, only two topical retinoids, tretinoin and tazarotene, have received U.S. Food and Drug Administration approval for treating photoaging [5]. The other anti-photoaging reagents available include numerous natural antioxidants such as ascorbic acid (Vt. C) and tocopherol (Vt. E), as well as medicinal plant extracts including polyphenolic compounds, particularly flavonoids [4,5]. For example, myricetin, a flavonoid found in several foods (i.e., onions, berries, grapes, and red wines), has shown anti-photoaging effects through its antioxidant and anti-inflammatory properties [6,7]. The beneficial effects have encouraged researchers to develop photo-protective products from natural sources [8,9,10,11].

Fucoidan, found mainly in marine brown algae, is a complex sulfated polysaccharide, which has various pharmacological properties including antioxidant, anti-inflammatory, anticoagulant, antiviral, and anticancer effects [12,13]. Furthermore, unlike native fucoidan with a high molecular-weight of approximately 20,000 kDa, low molecular-weight fucoidan (LMF)—less than 10 kDa—has shown more biological activities because of its high absorption and bioavailability [13]. However, a few studies have examined the anti-photoaging effect of fucoidans, which include three in vitro studies demonstrating the down-regulating effects on MMP-1 [14,15,16] and one in vivo study showing inhibitory effects on inflammation and MMP-1 expression following oral administration [17]. We previously showed that topical application of LMF has dermal wound healing effects with anti-inflammatory and antioxidant activities and modulates ECM rebuilding factors, such as transforming growth factor (TGF)-β1, fibroblast growth factor (FGF)-2, and MMPs [18]. It suggests that LMF also exerts biological effects involved in anti-photoaging.

To produce LMF, enzymatic hydrolysis methods are more advantageous than acid-hydrolysis or other conventional techniques; the method is non-toxic because enzymes are converted into water-soluble materials, and high bioactive compound yield and enhanced antioxidant activities are achieved [19]. Previously, fucoidan from Gamte, *Ecklonia cava* distributed along Korean coasts, has shown high antioxidant effects in a DPPH (1,1-diphenyl-2-picrylhydrazyl) free radical scavenging assay [19]. Therefore, we examined the anti-photoaging effects of LMF isolated from *E. cava*, using an enzymatic hydrolysis technique in UVB-irradiated mice, and the underlying mechanisms of these effects.

## 2. Results

### 2.1. Body Weight Changes

**Figure 1:**
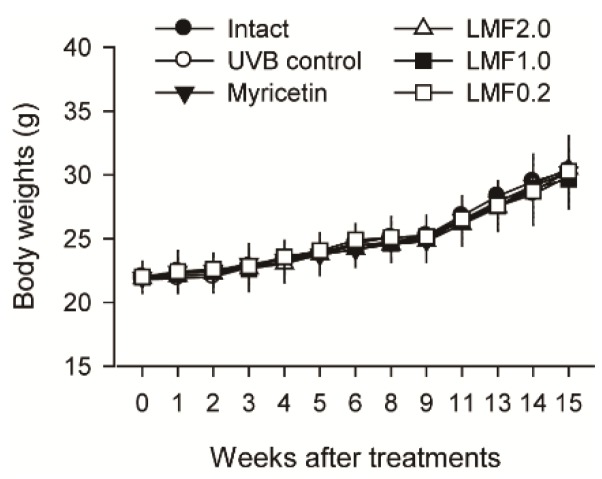
Non-irradiated normal mice and one group of UV (ultraviolet) B-irradiated mice were topically treated with distilled water (Intact and UVB control, respectively). The other four groups of UVB-irradiated mice were treated with myricetin (Myricetin) or low molecular-weight fucoidan (LMF) at 2.0, 1.0, and 0.2 mg/cm^2^ (LMF2.0, LMF1.0, and LMF0.2, respectively). The body weights were measured every week after treatment, and expressed as means ± SD of eight mice per group.

Body weights were normal in the UVB-irradiated mice (UVB control) compared with those of non-irradiated normal mice (Intact). The body weight changes did not differ among any groups regardless of the treatments (Figure 1).

### 2.2. Wrinkle Formation and Edema in UVB-Irradiated Skin

**Figure 2:**
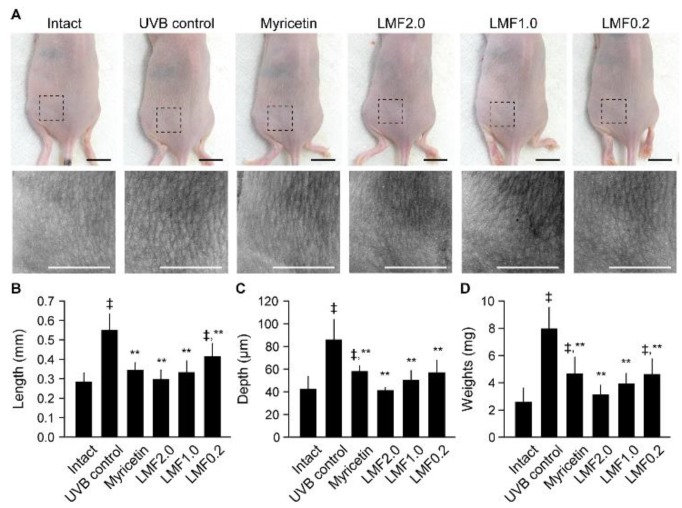
After treatments for 15 weeks, the left dorsal back skin (upper) and its replicas (lower) were observed (**A**). Scale bars indicate 10 mm. Wrinkle length (**B**) and depth (**C**) were assessed from the skin replicas. The dermal tissues were sampled and weighed (**D**). Values were expressed as means ± SD of 8 mice per group. ^‡^ *p* < 0.01 vs. intact and ** *p* < 0.01 vs. UVB control.

To examine the protective effects of LMF on photo-damages, skin wrinkle formation and tissue weights of the cutaneous edema were assessed. The UVB-irradiated dorsal back skin and its replicas showed evident wrinkle formation, but no skin cancer lesions were found. The wrinkle formation was observed to be severe in the UVB control group, whereas it was mild in the Myricetin and LMF groups (Figure 2A). Indeed, wrinkle length and depth in the skin replicas were significantly increased in the UVB control group compared with those in the intact, however, these values were decreased in the Myricetin and LMF groups compared with those in the UVB control (Figure 2B,C) (*p* < 0.05). In particular, the wrinkling degree did not differ between the LMF2.0 or LMF1.0 and the intact group. Wrinkle lengths were decreased by 37.4%, 46.1%, 39.5%, and 24.7% in the Myricetin, LMF2.0, LMF1.0, and LMF0.2 groups, respectively, and wrinkle depths were decreased by 32.5%, 52.3%, 41.3%, and 33.8%, respectively, compared with the corresponding values in the UVB control. Similarly, skin weight was significantly increased in the UVB control compared with that in the intact, but decreased in the Myricetin and LMF groups, especially in the LMF2.0 and LMF1.0 (Figure 2D) (*p* < 0.05). Decreases of 41.4%, 60.9%, 51.0%, and 42.1% in the Myricetin, LMF2.0, LMF1.0, and LMF0.2, respectively, were found compared with those in the UVB control group.

### 2.3. UVB-Irradiated Skin Inflammation

**Figure 3:**
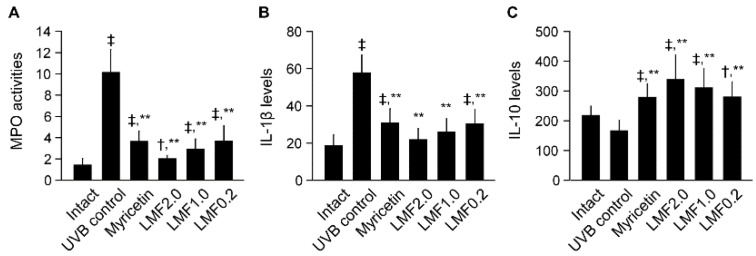
After treatments for 15 weeks, myeloperoxidase (MPO) activity (neutrophils × 10^5^ per mg of tissue protein, **A**) and skin levels of IL-1β and IL-10 (pg per 100 mg of protein, **B** and **C**, respectively) were assessed in UVB-irradiated skins. Values were expressed as means ± SD of 8 mice per group. ^‡^ *p* < 0.01, ^†^ *p* < 0.05 vs. intact, and ** *p* < 0.01 vs. UVB control.

UVB irradiation leads to leukocyte infiltration by dilating dermal blood vessels and increasing vascular hypermeability [8]. Thus, the skin myeloperoxidase (MPO) was assessed as a proinflammatory enzyme in the granulocytes. Consistently, the skin MPO activities were significantly increased in the UVB control compared with those in the intact (*p* < 0.05) (Figure 3A), indicating enhanced neutrophil recruitment to UVB-irradiated skin lesions. However, MPO activities were decreased in the Myricetin and LMF groups (*p* < 0.05). In addition, the dermal levels of IL-1β, a cytokine stimulating neutrophil, were increased in the UVB control compared with those in the intact, but decreased in the Myricetin and LMF groups compared with those in the UVB control (Figure 3B) (*p* < 0.05). In contrast, the level of IL-10, an anti-inflammatory cytokine, was significantly increased in the Myricetin and LMF groups compared with that in the UVB control (Figure 3C) (*p* < 0.05).

### 2.4. Antioxidant Activities in UVB-Irradiated Skin

The skin contents of glutathione (GSH) as an endogenous antioxidant were measured, and level of malondialdehyde (MDA) for lipid peroxidation and superoxide anion were assessed for the oxidative stress (Table 1). The GSH content was significantly decreased in the UVB control compared with that in the intact, but increased in the Myricetin and LMF groups (*p* < 0.05). In contrast, the levels of MDA and superoxide anion were significantly increased in the UVB control compared with those in the intact, but decreased in the Myricetin and LMF groups, particularly in the LMF2.0 (*p* < 0.05). The higher GSH and the lower levels of MDA and superoxide suggest antioxidant effects of LMF on the UVB-damaged skins. The antioxidant activities were further examined by measuring the mRNA expression levels of GSH reductase, an enzyme that regenerates GSH from the oxidized disulfide form, and Nox2, a nicotinamide adenine dinucleotide phosphate (NADPH) oxidase related to ROS formation (Table 2). The mRNA expression of GSH reductase was significantly lower in the UVB control than in the intact, but higher in the Myricetin and LMF groups than in the UVB control (*p* < 0.05). In contrast, Nox2 expression was higher in the UVB control than in the intact, but lower in the Myricetin and LMF groups than in the UVB control (*p* < 0.05).

### 2.5. mRNA Expression of MMPs Related to Skin Photoaging

As UV radiation stimulates MMPs to promote the breakdown of collagen [20], the skin remodeling process was examined by detecting the mRNA expression of MMP-1, MMP-9, and MMP-13 (Table 2). These expressions were significantly up-regulated in the UVB control compared with that in the intact (*p* < 0.05). However, they were significantly down-regulated in the Myricetin and LMF groups compared with that in the UVB control (*p* < 0.05).

### 2.6. Histopathological Changes

**Figure 4:**
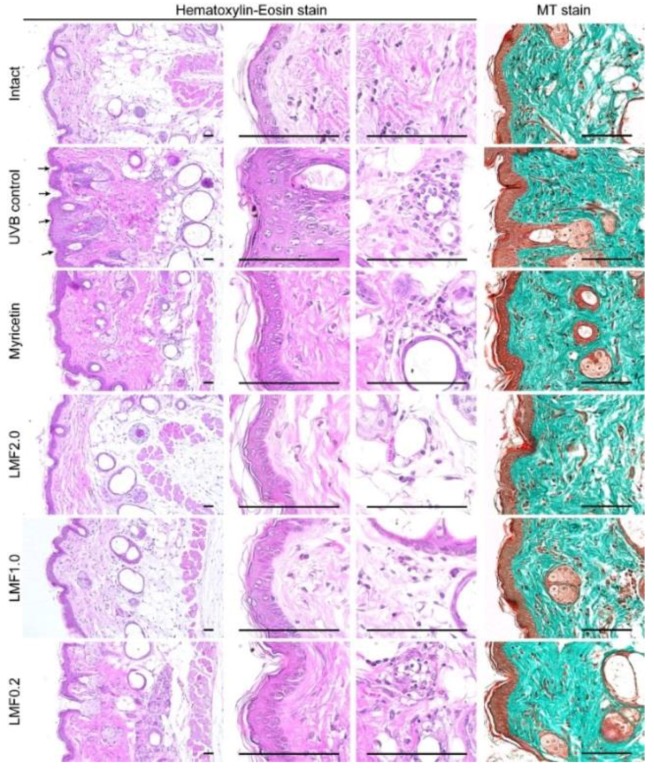
Skin tissue sections were stained with hematoxylin and eosin or Masson’s trichrome (MT). Arrows indicate epithelial microfolds formed. Scale bars indicate 100 μm.

The UVB control exhibited increased epithelial thickness and microfold formation with hyperplasia and hypertrophy of the epidermal keratinocytes in hematoxylin-eosin stains (Figure 4). In addition, the UVB control showed increases in altered collagen deposition in the Masson’s trichrome (MT) stains. However, the histopathological changes appeared to be reversed in the Myricetin and LMF groups. Histomorphometric analyses revealed significant increases in epithelial thickness, microfolds, infiltrated inflammatory cells, and regions occupying collagen fibers in the UVB control compared with those in the intact (*p* < 0.05) (Table 3). However, the changes were significantly inhibited in the Myricetin and LMF groups compared with those in the UVB control (*p* < 0.05).

### 2.7. Immunohistochemistry

**Figure 5:**
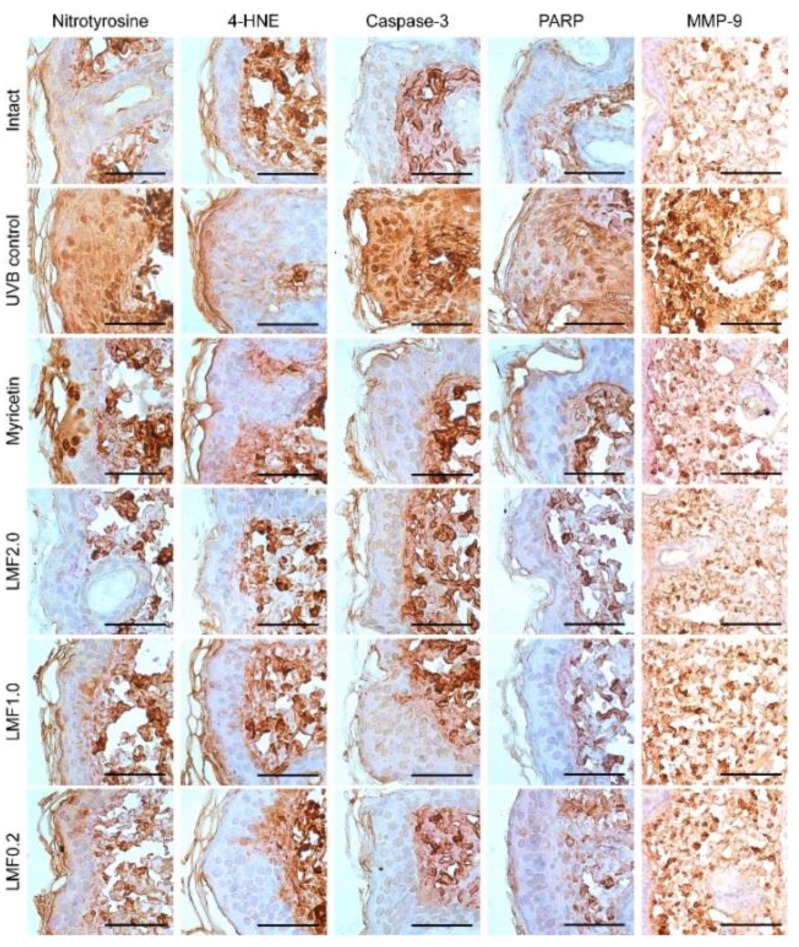
Skin tissue sections were immunostained for nitrotyrosine and 4-hydroxynonenal (4-HNE), as markers of oxidative stress; caspase-3 and poly (adenosine diphosphate-ribose) polymerase (PARP) as markers of apoptosis; and matrix metalloproteinase (MMP)-9. Next, the sections were counterstained with hematoxylin. Scale bars indicate 50 μm.

Immunostaining for nitrotyrosine and 4-hydroxynonenal (4-HNE), as markers of oxidative stress, and caspase-3 and cleaved poly (adenosine diphosphate-ribose) polymerase (PARP), as markers of apoptosis, showed more intense signals in the UVB control than in the intact. The MMP-9 was also detected more in the UVB control than the intact (Figure 5). However, the tendencies were decreased in the Myricetin and LMF groups. Histomorphometric analyses revealed significant increases in immunoreactive cells for nitrotyrosine, 4-HNE, caspase-3, PARP, and MMP-9 in the UVB control compared with those in the intact (*p* < 0.05) (Table 4). However, the immunoreactive cells were significantly reduced in the Myricetin and LMF groups (*p* < 0.05).

## 3. Discussion

Similar to the clinical symptoms of chronic UV exposure, our UVB-irradiated model exhibited increased winkle formation and dermal thickness, and decreased skin elasticity [1]. However, LMF treatment inhibited photoaging by enhancing antioxidant, anti-inflammatory, and anti-apoptotic activities and inhibiting ECM degradation through down-regulating UV-responsive genes encoding MMP-1, MMP-9, and MMP-13. Because the LMF treatment was mostly absorbed before the irradiation, the results seemed to be involved in photo-protective effects rather than UV filtering effects. Although only one in vivo study has demonstrated the anti-photoaging effects of native fucoidan via oral administration [17], this is the first study to show that LMF ameliorates UBV-induced photoaging via topical application.

UVB irradiation induces ROS production and deteriorates the antioxidant defense system, leading to a state of oxidative stress [21]. Here, UVB irradiation up-regulated the mRNA expression of Nox2 as an ROS producer and increased the dermal contents of superoxide anion and MDA, while it reduced dermal GSH contents by down-regulating GSH reductase. However, LMF treatment inhibited the progression to oxidative stress and enhanced innate antioxidant activities. Immunostaining for nitrotyrosine or 4-HNE also revealed that LMF exert a strong antioxidant activity against photo-damage. To date, fucoidans from various algae, including *Porphyra haitanesis*, *Ulva pertusa*, *F. vesiculosus*, *Laminaria japonica*, and *Ecklonia kurome* have been shown to possess antioxidant properties [13]. The beneficial effects are thought to be conferred by the chemical compositions of fucoidans such as sulfate, monosaccharide, sugar residue [18,22]. It suggests that LMF has substantial fucose and sulfate content as a natural antioxidants involved in modulating a number of oxidative stress-mediated diseases including photoaging.

With the antioxidant activities, LMF treatment showed anti-inflammatory effects on UVB-irradiated skins, which was supported by inhibiting edema and neutrophil recruitment to photo-damaged lesions. The anti-inflammatory activities of LMF may contribute to decreasing IL-1β and increasing IL-10 levels. The imbalance between ROS production and the antioxidant defense system is known to cause skin inflammation through complex pathways [23]. ROS activate mitogen-activated protein kinase (MAPK) signaling transduction pathways. MAPK pathways activate nuclear factor (NF)-κB and activator protein-1 (AP-1), which enhances the release of inflammatory cytokines such as tumor necrosis factor-α, IL-1β, IL-6, and IL-8 [21,24]. In particular, Nox2 and superoxide anion contribute to stimulating neutrophil infiltration. In this context, the antioxidant effects of LMF may result in inhibition of the inflammatory progress. However, the anti-photoaging effects of LMF likely occur through interactive mechanisms between antioxidant and anti-inflammatory effects. For example, because fucoidan directly inhibits neutrophil infiltration by blocking selectin [25], the reduced neutrophils may be linked to inhibition of the release of free oxygen radicals by reducing MPO activities. In addition, because IL-1β activates Nox complexes as producers of ROS and IL-10 inhibits the NF-κB pathways, the decreased IL-1β and increased IL-10 may be linked to inhibited production of ROS and further release of inflammatory cytokine [26]. Thus, the antioxidant and anti-inflammatory effects of LMF have therapeutic potentials for treating skin photoaging.

Wrinkle formation is closely related to the degradation of ECM proteins via collagen fragmentation and MMP secretion [20]. MMPs are activated by excessive oxidative stress or inflammatory responses: oxidative stresses up-regulate MMPs including MMP-1, MMP-3, MMP-9, and MMP-13 through binding of AP-1 to MMPs, and pro-inflammatory cytokines also up-regulate MMPs and degrade dermal collagen elastin fibers [27]. Thus, the development of MMP inhibitors is considered a promising strategy for anti-photoaging. Some flavonoid compounds, such as naringenim, apigenin, wogonin, kaempferol, and quercetin, have been reported to inhibit the expression of MMP-1 and type I procollagen [28]. Here, LMF treatment down-regulated the gene expression of MMP-1, MMP-9 and MMP-13. Previous studies have shown that native fucoidans modulate MMP-1 expression in human fibroblasts [14,15,16]. Furthermore, l-fucose and fucose-rich polysaccharides have a direct relationship with increased synthesis of elastin and collagen by down-regulating MMPs, particularly MMP-2 and MMP-9 [29,30,31]. These results demonstrate that LMF attenuates connective tissue damage by inhibiting MMP activities and enhancing collagen synthesis.

In photoaging, a failure in the repair mechanisms of DNA damage leads to apoptosis through the AP-1 signaling pathway [32]. Various molecules, such as T4N5, photolyase, and thymidine dinucleotide, have been proven to be valuable for photoaging protection by enhancing the repair of DNA photo-damage [5]. Fucoidans isolated from various brown algae also have been reported to inhibit oxidative DNA damage in tumor cells [33,34] and diabetic cardiomyocytes [35]. The beneficial effects are involved in the interactions between fucoidans and growth factors including basic FGF [36] and TGF-β [37], suggesting their therapeutic potentials for tissue repair. Indeed, LMF decreased immunoreactive cells for caspase-3 and PARP in UVB-irradiated skin lesions, likely by inhibiting the MAPK pathways related to NF-κB and AP-1.

Overall, these results demonstrate the anti-photoaging effects of LMF on UVB-irradiated skin damage by cooperative interactions of antioxidant, anti-inflammatory, and MMP-inhibiting effects. Furthermore, fucoidan has been reported to inhibit melanin formation, which may be useful for developing treatments for hyperpigmentation [38]. Taken together, these finding suggest that LMF can serve as a potential agent for treating UV-related skin disease.

## 4. Materials and Methods

### 4.1. Reagents

LMF was kindly provided by Glucan Corp. Ltd. (Busan, Korea). To produce LMF, a commercial high molecular-weight fucoidan from Gamte, *E. cava* (Aqua Green Technology Co., Ltd., Jeju, Korea) was reduced by reacting fucoidan with fucoidanase isolated from *Pseudoalteromonas* sp. (strain 1493) for 2 h at 50 °C and pH 8 [9,39]. The resulting solution was filtered through 10 and 5 kDa ceramic membranes, and then lyophilized. The molecular weight was nearly 8 kDa according to gel permeation chromatography based on high-performance liquid chromatography analysis. Myricetin was purchased from Sigma-Aldrich (St. Louis, MO, USA). LMF and myricetin were dissolved in distilled water and acetone, respectively.

### 4.2. Animals

All animal experiments were performed according to the national regulations of the usage and welfare of laboratory animals and approved by the Institutional Animal Care and Use Committee in Daegu Haany University (Gyeongsan, Korea) (DHU2012-058, 20 October 2012). Six-week female HR-1 hairless mice (SLC, Shizuoka, Japan) were housed in a polycarbonate cage and maintained in a temperature (20–25 °C) and humidity (50–55%) controlled room with a 12-h light/dark cycle. Food and water were supplied ad libitum.

### 4.3. Skin Photoaging Model and Treatment

After eight day acclimatization, the mice were divided into six groups (*n* = 8/group) with similar body weights. In five groups, skin photoaging was induced by 0.18 J/cm^2^ UVB irradiation three times a week using a UV crosslinker system emitting wavelengths of 254 nm, 312 nm, and 365 nm, with a peak emission at 312 nm (Hoefer Scientific Instruments, San Francisco, CA, USA), as described previously [40]. The remaining group was not irradiated with the off-system. Treatment was applied topically to the left dorsal back skin in a 1 × 1 cm area near the gluteal region in a volume of 200 μL as follows: the UVB non-irradiated group and one irradiated group were treated with distilled water (intact and UVB control, respectively), and other UVB-irradiated groups were treated with myricetin at 5 nM (0.32 ng/cm^2^) (Myricetin) or LMF at 10 (2.0 mg/cm^2^), 5 (1.0 mg/cm^2^), and 1 mg/mL (0.2 mg/cm^2^) (LMF2.0, LMF1.0, and LMF0.2, respectively). The application was performed once a day for 15 weeks. The body weight of mice was measured every week.

### 4.4. Macroscopic Analysis of UVB-Irradiated Skin

UVB-irradiated skin wrinkles were assessed in replicas of the mouse dorsal skins including the treated region using the Repliflo Cartridge Kit (CuDerm Corp., Dallas, TX, USA), as described previously [40]. Wrinkle shadows of the impression replicas were generated using an optical light with an angle of 40°. The black and white images were analyzed by Skin-Visiometer VL650 software (Courage & Khazaka, Cologne, Germany). Next, the dorsal skin was sampled using a punch with a 6-mm diameter, and the sample was weighed for skin edema. The skin samples, including other treated regions, were homogenized for biochemical analyses or fixed for histopathological analyses.

### 4.5. Measurement of Leukocyte Migration to UVB-Irradiated Skin

Leukocyte migration to UVB-irradiated skin damage was analyzed by the MPO assay [24]. The skin sample was homogenized in 50 mM K_2_HPO_4_ buffer (pH 6.0) containing 0.5% hexadecyltrimethylammonium bromide for 15 s on ice. After centrifuging at 1000× *g* for 2 min at 4 °C, the supernatants were mixed with the K_2_HPO_4_ buffer (pH 6.0) containing 0.167 mg/mL o-dianisidine dihydrochloride and 0.05% hydrogen peroxide, and the absorbance was assessed at 450 nm (OPTIZEN POP, Mecasys, Daejeon, Korea). The tissue protein was measured using the Lowry method. In comparison with a standard curve of neutrophils, MPO activity was expressed as the number of neutrophils/mg of protein.

### 4.6. Measurement of IL-1β and IL-10 in UVB-Irradiated Skin

The skin samples were homogenized as described previously [40]. IL-1β and IL-10 was assessed using an enzyme-linked immunosorbent assay kit (Abcam, Cambridge, UK) according to the manufacturer’s instructions. The absorbance was measured at 490 nm using a microplate spectrophotometer reader (Tecan, Männedorf, Switzerland).

### 4.7. Antioxidant Activities in UVB-Irradiated Skin

To determine glutathione (GSH) contents, skin sample was homogenized in 100 mM NaH_2_PO_4_ buffer solution (pH 8.0) containing 5 mM ethylenediaminetetraacetic acid (1:3, *w*/*w* dilution). The homogenates were added with 30% trichloroacetic acid, and centrifuged twice at 1940× *g* for 6 min and then at 485× *g* for 10 min. The supernatant was added to 1 mg/mL o-phthalaldehyde (Sigma-Aldrich), and measured in a fluorescence spectrophotometer (RF-5301PC; Shimadzu Corp., Tokyo, Japan) (kexc = 350 nm; kem = 420 nm). Values were expressed as μM of GSH/mg of protein compared with a standard curve using diluted solutions of GSH (75 μM). Another tissue sample was homogenized at 10 mg/mL in 1.15% KCl, as described previously [40]. For MDA, the homogenates were added to 10% trichloroacetic acid, and centrifuged at 1000× *g* for 3 min. The supernatant was incubated with 0.67% thiobarbituric acid for 15 min at 100 °C, and then assessed at 535 and 572 nm using a spectrophotometer reader (Tecan). For superoxide anion, the homogenates were incubated with 1 mg/mL nitroblue tetrazolium (NBT, Sigma-Aldrich) for 1 h at 37 °C. The supernatant was removed and the formazan precipitates were solubilized with a mixture of 2 M potassium hydroxide and dimethyl sulfoxide. The reduction of NBT to formazan by superoxide anion was measured at 600 nm using a spectrophotometer reader (Tecan).

### 4.8. Quantitative Reverse Transcription Polymerase Chain Reaction (qRT-PCR) Analysis

Total skin tissue RNA was extracted using TRIzol reagent (Invitrogen, Carlsbad, CA, USA), as described previously [24,40]. RNA concentration and quality were analyzed using a CFX96^TM^ Real-Time System (Bio-Rad, Hercules, CA, USA). The sample was treated with recombinant DNase I (DNA-free; Ambion, Austin, TX, USA) to remove contaminating DNA, and RNA was reverse-transcribed using a reagent High-Capacity cDNA Reverse Transcription Kit (Applied Biosystems, Foster City, CA, USA) according to the manufacturer’s instructions. A total of 50 PCR cycles were performed as follows: 95 °C for 15 s, 60 °C for 20 s, and 72 °C for 30 s for denaturation, annealing, and extension, respectively. The primers used are listed in Table S1.

### 4.9. Histopathology

Skin samples were fixed in 10% neutral buffered formalin. The samples were paraffin-embedded and sectioned at a thickness of 3 μm. The sections were stained with hematoxylin and eosin (H&E) or Masson’s trichrome (MT). In H&E, histomorphometric analyses were performed for epithelial microfolds (folds/mm of epithelium), epithelial thicknesses (μm), and inflammatory cells infiltrated in the dermis (cells/mm^2^ of dermis), using a computer-assisted image analysis program (iSolution FL ver 9.1, IMT i-solution Inc., Vancouver, BC, Canada). In the MT stain, the area occupying collagen fiber (%/mm^2^ of dermis) was assessed. The histopathologist was blinded to the treatment groups.

### 4.10. Immunohistochemistry

The other serial sections were deparaffinized and rehydrated, followed by antigen retrieval pretreatment in 10 mM citrate buffer for 20 min at 95–100 °C (Shi et al., 1993). The sections were immunostained using a Vectastain Elite ABC Kit (Vector Lab., Inc., Burlingame, CA, USA), as described previously [18,40]. Briefly, endogenous peroxidase was inactivated by 0.3% H_2_O_2_ for 30 min, and non-specific binding of proteins was blocked with normal horse serum for 1 h. The sections were incubated with primary rabbit polyclonal antibodies for cleaved caspase-3 (# 9661, Cell Signaling Technology Inc., Danvers, MA, USA, 1:400), cleaved PARP (# 9545, Cell Signaling Technology, 1:100), 4-HNE (# Ab 46545, Abcam, 1:100), nitrotyrosine (# 06-284, Millipore, Billerica, MA, USA, 1:200), or MMP-9 (# Ab 38898, Abcam, 1:100), overnight at 4 °C. The following day, the sections were incubated with biotinylated secondary antibody and then ABC reagents for 1 h each. Immunoreactivity was visualized using a peroxidase substrate kit (Vector Lab.) for 3 min, and counterstained with hematoxylin. All incubation procedures were carried out in a humidity chamber, and the sections were rinsed with 10 mM phosphate-buffered saline three times between each step. Cells or fibers occupying more than 30% of the immunoreactivity were regarded as positive, and analyzed using the iSolution program. The histopathologist was blinded to the groupings.

### 4.11. Statistical Analyses

Values are expressed as means ± standard deviation (SD) of eight sample sizes. Variance homogeneity was examined by using the Levene test. If no significance was detected, the data were analyzed by one way analysis of variance (ANOVA) followed by a least-significant differences multi-comparison (LSD) post hoc test. In a case of significances, a non-parametric Kruskal–Wallis H test was conducted, followed by the Mann–Whitney U (MW) post hoc test. The analyses focused mainly on the differences among treatment groups compared with the UVB control. A *p*-value < 0.05 indicated significance.

## Figures and Tables

**Table 1:** Antioxidant activities in ultraviolet (UV) B-irradiated skins.

	GSH (μM/mg)	MDA (nM/mg)	Superoxide Anion (NBT Reduction)
Intact	1.47 ± 0.62	0.34 ± 0.11	0.36 ± 0.08
UVB control	0.41 ± 0.09 ^‡^	1.68 ± 0.41 ^‡^	1.03 ± 0.18 ^‡^
Myricetin	0.95 ± 0.15 ^†^^,^**	0.69 ± 0.20 ^‡^^,^**	0.56 ± 0.16 ^‡^^,^**
LMF2.0	1.44 ± 0.14 **	0.40 ± 0.10 **	0.36 ± 0.08 **
LMF1.0	1.36 ± 0.20 **	0.51 ± 0.13 ^†^^,^**	0.49 ± 0.08 ^†^^,^**
LMF0.2	0.94 ± 0.18 ^†^^,^**	0.67 ± 0.13 ^‡^^,^**	0.56 ± 0.15 ^‡^^,^**

Non-irradiated normal group and one group of UVB-irradiated mice were topically treated with distilled water (intact and UVB control, respectively). The other four groups of UVB-irradiated mice were treated with myricetin at 0.32 ng/cm^2^ (Myricetin) or low molecular-weight fucoidan (LMF) at 2.0, 1.0, and 0.2 mg/cm^2^ (LMF2.0, LMF1.0, and LMF0.2, respectively). After treatment for 15 weeks, the glutathione (GSH), malondialdehyde (MDA) and superoxide anions were assessed in the skin tissues and they were normalized to the tissue proteins. Values are expressed as means ± SD of eight mice. NBT = nitroblue tetrazolium. ^‡^: *p* < 0.01 and ^†^: *p* < 0.05 vs. intact, and **: *p* < 0.01 vs. UVB control.

**Table 2:** Tissue mRNA expressions in UVB-irradiated skins.

	MMP-1	MMP-9	MMP-13	GSH Reductase	Nox2
Intact	1.04 ± 0.09	1.07 ± 0.10	1.06 ± 0.07	1.01 ± 0.09	1.01 ± 0.08
UVB control	2.02 ± 0.20 ^‡^	1.93 ± 0.23 ^‡^	2.29 ± 0.25 ^‡^	0.79 ± 0.15 ^‡^	1.71 ± 0.22 ^‡^
Myricetin	1.34 ± 0.18 ^‡^^,^**	1.31 ± 0.14 ^‡^^,^**	1.47 ± 0.21 ^‡^^,^**	1.28 ± 0.16 ^‡^^,^**	1.22 ± 0.14 ^‡^^,^**
LMF2.0	1.06 ± 0.09 **	1.12 ± 0.08 **	1.15 ± 0.07 ^†^^,^**	1.93 ± 0.19 ^‡^^,^**	1.06 ± 0.08 **
LMF1.0	1.20 ± 0.09 ^†^^,^**	1.16 ± 0.09 ^†^^,^**	1.26 ± 0.05 ^‡^^,^**	1.58 ± 0.36 ^‡^^,^**	1.12 ± 0.08 ^†^^,^**
LMF0.2	1.33 ± 0.14 ^‡^^,^**	1.28 ± 0.09 ^‡^^,^**	1.47 ± 0.17 ^‡^^,^**	1.30 ± 0.12 ^‡^^,^**	1.22 ± 0.05 ^‡^^,^**

After treatment for 15 weeks, expressions of mRNA for matrix metalloprotease (MMP)-1, -9, and -13, GSH reductase and nicotinamide adenine dinucleotide phosphate (NADPH) oxidase 2 (Nox2) were assessed. Values are expressed as means ± SD of eight mice for relative mRNA expressions per β-actin. ^‡^: *p* < 0.01, ^†^: *p* < 0.05 vs. intact, and **: *p* < 0.01 vs. UVB control.

**Table 3:** Histopathological changes on UVB-irradiated skins.

	Microfolds (Folds/mm)	Epi. Thickness (μm)	IF Cells (Cells/mm^2^)	Collagen Fiber (%/mm^2^)
Intact	10.50 ± 3.63	20.54 ± 2.52	9.50 ± 2.98	45.31 ± 5.75
UVB control	74.75 ± 10.94 ^‡^	48.28 ± 5.04 ^‡^	269.50 ± 50.65 ^‡^	82.54 ± 8.20 ^‡^
Myricetin	39.50 ± 13.73 ^‡^^,^**	30.26 ± 5.08 ^‡^^,^**	204.50 ± 51.29 ^‡^^,^*	59.32 ± 6.17 ^‡^^,^**
LMF2.0	19.63 ± 4.17 ^‡^^,^**	26.75 ± 4.04 ^‡^^,^**	31.00 ± 7.19 ^‡^^,^**	48.44 ± 2.97 **
LMF1.0	38.63 ± 5.10 ^‡^^,^**	30.63 ± 2.04 ^‡^^,^**	61.25 ± 14.57 ^‡^^,^**	55.72 ± 7.89 ^‡^^,^**
LMF0.2	56.88 ± 5.99 ^‡^^,^**	32.89 ± 3.83 ^‡^^,^**	140.00 ± 38.37 ^‡^^,^**	59.77 ± 8.94 ^‡^^,^**

After treatments for 15 weeks, microfolds, epithelial (Epi.) thickness, and inflammatory (IF) cells were assessed in hematoxylin-eosin stains in Figure 4, and relative regions of collagen fiber was assessed in Masson’s trichrome stains. Values are expressed as means ± SD of eight mice. ^‡^: *p* < 0.01 vs. intact, **: *p* < 0.01, and *: *p* < 0.05 vs. UVB control.

**Table 4:** Immunohistochemistry in UVB-irradiated skins.

	Nitrotyrosine	4-HNE	Caspase-3	PARP	MMP-9
Intact	12.00 ± 4.21	12.50 ± 3.07	17.38 ± 3.25	15.13 ± 3.04	31.25 ± 8.58
UVB control	86.88 ± 10.11 ^‡^	78.00 ± 10.72 ^‡^	83.50 ± 10.17 ^‡^	79.13 ± 11.00 ^‡^	81.75 ±11.62 ^‡^
Myricetin	50.00 ± 6.70 ^‡^^,^**	43.13 ± 12.17 ^‡^^,^**	32.00 ± 6.55 ^‡^^,^**	38.88 ± 8.01 ^‡^^,^**	51.88 ± 8.04 ^‡^^,^**
LMF2.0	21.38 ± 4.44 ^‡^^,^**	19.50 ± 2.56 ^‡^^,^**	21.13 ± 2.90 ^†^^,^**	21.13 ± 2.53 ^‡^^,^**	43.00 ± 5.13 ^‡^^,^**
LMF1.0	36.25 ± 5.65 ^‡^^,^**	33.00 ± 3.70 ^‡^^,^**	26.13 ± 4.22 ^‡^^,^**	23.88 ± 3.14 ^‡^^,^**	48.63 ± 5.01 ^‡^^,^**
LMF0.2	50.25 ± 6.11 ^‡^^,^**	39.75 ± 4.53 ^‡^^,^**	30.25 ± 3.20 ^‡^^,^**	36.75 ± 5.01 ^‡^^,^**	53.00 ± 8.02 ^‡^^,^**

After treatments for 15 weeks, immunostains in Figure 5 were examined; immunostains for nitrotyrosine, 4-hydroxynonenal (4-HNE), and caspase-3 and cleaved poly (adenosine diphosphate-ribose) polymerase (PARP) were assessed as epidermal immunoreactive cells per 100 epithelial cells, and matrix metalloprotease (MMP)-9 was assessed as relative immunoreactive regions per regions of interests (%). Values are expressed as means ± SD of eight mice. ^‡^: *p* < 0.01, ^†^: *p* < 0.05 vs. intact, and **: *p* < 0.01 vs. UVB control.

## Supplementary Materials

The following are available online at http://www.mdpi.com/1660-3397/16/8/286/s1, Table S1: Primers used for quantitative RT-PCR.
