# Supplementary material for: Anti-Photoaging Effects of Low Molecular-Weight Fucoidan on Ultraviolet B-Irradiated Mice
Source: Mar Drugs. 2018 Aug 18;16(8):286. doi: 10.3390/md16080286 (PMC6117676; doi:10.3390/md16080286)
Supplement: Supplementary file 1 [file marinedrugs-16-00286-s001.pdf]

marine drugs

MDPI

Table S1. Primers used for quantitative RT-PCR

Targets	Sequence
MMP-1	Forward : 5′-AAG GTT AGC TTA CTG TCA CAC GCT T-3′<br>Reverse : 5′-CGA CTC TAG AAA CAC AAG AGC AAG A-3′
MMP-9	Forward : 5′-CCC GGA CCA AGG ATA CAG-3′<br>Reverse : 5′-GGC TTT CTC TCG GTA CTG-3′
MMP-13	Forward : 5′-CAT CCA TCC CGT GAC CTT AT-3′<br>Reverse : 5′-GCA TGA CTC TCA CAA TGC GA-3′
GSH reductase	Forward : 5′-TGC GTG AAT GTT GGA TGT GTA CCC-3′<br>Reverse : 5′-CCG GCA TTC TCC AGT TCC TCG-3′
Nox2	Forward : 5′-AGC TAT GAG GTG GTG ATG TTA GTG G-3′<br>Reverse : 5′-CAC AAT ATT TGT ACC AGA CAG ACT TGA G-3′
β-actin	Forward : 5′-AGC TGC GTT TTA CAC CCT TT-3′<br>Reverse : 5′-AAG CCA TGC CAA TGT TGT CT-3′
